# Prevalence of sexual abuse of male high school students in Addis Ababa, Ethiopia

**DOI:** 10.1186/1472-698X-13-24

**Published:** 2013-05-16

**Authors:** Rahel Tesfaye Haile, Negussie Deyessa Kebeta, Getnet Mitike Kassie

**Affiliations:** 1 TSEHAI project, Johns Hopkins University, Addis Ababa, Ethiopia; 2 School of Public Health Addis Ababa University, Addis Ababa, Ethiopia

**Keywords:** Sexual abuse, Rape, Male students

## Abstract

**Background:**

Sexual abuse of boys is a neglected problem in many developing countries including Ethiopia. As a result, its prevalence, contributing factors and circumstance in which sexual abuse occurs are largely unknown. The objective of this study is to determine the prevalence and factors associated with sexual abuse of male high school students in Addis Ababa city administration.

**Methods:**

A cross-sectional descriptive study involving 884 randomly selected students of nine high schools in Addis Ababa was conducted in March 2009. A pre-tested questionnaire was used to collect data. Analysis of the data was made using SPSS for windows version 15.

**Results:**

This study indicates the life time prevalence of rape and sexual harassment of boys in Addis Ababa were 4.3%, and 68.2%, respectively. The chance of experiencing sexual coercion was higher among students who live alone (AOR = 2.87; 95% CI; 1.07, 7.66) and among students who live with others (AOR =1.80; 95% CI = 1.04, 3.11) than those living with both parents. Similarly, the odds of experiencing rape in their life time was higher among students who live with others (AOR=2.20; 95% CI; 1.04, 4.68) than those who live with their parents.

**Conclusions:**

Sexual abuse of male students is not uncommon in Addis Ababa. It is higher in those living alone or not living with their parents. Due attention is needed by schools, parents and other concerned bodies. Designing a program to fight against sexual abuse should include young school boys.

## Background

Researchers do not have the same definition for sexual abuse [1]. Sexual abuse includes attempted or completed sexual intercourse (i.e., oral, anal, vaginal); touching, kissing, or rubbing up; exhibiting their body parts; or having any type of sex act committed by an adult or under age [2]. Sexual abuse of girls has been widely studied; however, data in regards to sexual abuse of boys including its prevalence and associated factors have remained limited. One of the major challenges of studying sexual abuse of males is underreporting due to fear of consequences and resulting social stigma. Underreporting is related to many issues. For instance, boys are less likely than girls to report sexual abuse because of fear, social stigma against homosexual behavior, and the desire to appear self-reliant (boys grow up believing that they should not allow themselves to be harmed or talk about painful experiences related to sexual vulnerability) [3].

The prevalence estimates of sexual abuse of boys vary widely (ranging from 4%-76%) [1], which is as a result of differences in study definition, design and methods. Approximately one in six boys is sexually abused before the age of 16.2 years [1,3]. In the 1999 Regional Office consultative meeting, participants from 28 countries representing all the African sub-regions reported that sexual abuse is a serious concern in their countries [4], there is an enormous burden of sexual violence and harassment in secondary schools, with both boys and girls experiencing some form of sexual abuse [5].

There are several consequences of sexual abuse of boys including physical, psychological and social problem, and the consequence can be immediate as constraining their educational performance, risk to Human Immune Deficiency Virus (HIV), Sexual Transmitted Infections (STI) and the late consequence could be social isolation, fear, phobia and hopelessness.

Ethiopia is one of the least developing countries with multiethnic and diverse socio-cultural background where the majority (84%) of the population lives in rural areas. Most of the populations are followers of the Orthodox Christian and Muslim religions. It is a male dominated society with deep-rooted issues of gender including defining roles of men and women. In most Ethiopian cultures, sex is something that is not discussed openly. Therefore public knowledge towards sexual abuse of boys is very low compared to sexual violence against girls. Addis Ababa a rapidly growing city serving as the Capital; city of Ethiopia as well as the seat of African Union and many diplomatic corps. There are limited reports on sexual abuse of boys and most are done by nongovernmental organizations. For instance, Forum on Street Children-Ethiopia (FSCE) has reported on sexual abuse of boys for creating public awareness. A two-year reported data by the police from all sub-cities of Addis Ababa indicated that there is a considerable number of reported male victims of rape in Addis Ababa [6]. The objective of this study is therefore to determine the prevalence and identify its contributing factors of sexual abuse of male high school students in Addis Ababa city administration and there by create public awareness for action.

## Methods

The study was conducted in July 2009 in Addis Ababa, the capital city of Ethiopia. The population is about 2,739,551 [7]. The total number of students in all senior secondary school at the time of the study was 49,038, of which 24,158 (49.3%) were males [8].

### Design

School -based descriptive cross-sectional study was conducted among randomly selected male senior secondary students [Preparatory (Grade 11–12)] in Addis Ababa.

### Study participants

The source population for this study was all senior secondary school students found in Addis Ababa city administration including public and private. The sample included all senior male students who were registered in regular programs in the selected senior secondary schools and fulfilled the inclusion criteria. The study did not include male students attending evening classes due to logistic problems.

#### Inclusion criteria

Regular (day time) senior secondary schools students Grade-11 and Grade-12, of age 18 years and above who were present in the school on the day of the administration of the questionnaire were included.

#### Exclusion criteria

Students who were not able to complete the questionnaire without assistance due to visual impairment, language barrier, foreign community schools and students unwilling to participate in the study were excluded. Students <18years of age were excluded because of the legal definition of children in Ethiopia which is under 18 years of age. Moreover, consent from a parent or a guardian was not possible because of the sensitiveness of the study questions and possible negative implications.

### Sample size and sampling procedure

Sample size was calculated considering 50% prevalence of sexual abuse among boys, 95% certainty and 5% margin of error between population and sample with a non response rate (15%) and design effect of 2 for the multistage sampling. Hence the total sample size calculated for this study was 884. A list of schools was prepared in accordance to ownership including government, private and others categorized into three strata. Accordingly, 57 schools were stratified by ownership type: Government = 10 [total population of students =12598 in 116 sections], Private = 28 [total population of students= 3853 in 41 sections] and others= 19 / non-governmental organization including missionaries and religious organizations [total population of students =7707 in 26 sections). Probability to population size was calculated for each selected school to obtain the total calculated sample size for each stratum. In order calculate the sample size of each stratum, population size of the stratum was multiplied by the total sample size of the strata and divided by the total student population (24158).

Multi stage sampling was used to select schools, sections and students from each stratum. First, using simple random sampling method three senior secondary schools were selected from each stratum (a total of nine). One private school did not agree to participate in the study and was replaced by another school using simple random sampling. Second, from the selected schools, all sections were listed and then using simple random sampling the sections that were included in the study were selected. Third, a separate list of boys in the selected sections was developed by unit leaders of the sections and then eligible boys were recruited from the list of male students using simple random sampling method.

### Data collection

A structured questionnaire adapted from the WHO multi-country core questionnaire on violence against women and other internationally developed questionnaires on sexuality was used [9]. The questionnaire has sections on socio-demographic characteristics of participants, family history including educational background of parents and sexuality of boys.

The following are examples of questions that respondents were asked: With whom are you currently living? Do your mother and father live together? With whom you slept together in your home? Have you ever had sexual intercourse? What made you have first sexual intercourse? What type of sexual intercourse did you have? What initiated you to have sex with a male partner? Who was the perpetrator? If you escaped forced sex, how did you mange the attempt? If you encountered forced sex where was the place? After the attack to who did you share the problem? If you did not report to anybody, why did you keep it secret? Which of the following did you experience after forced sex? Have you ever used the following drugs? Alcohol, Khat, drugs like Cannabis, cigarettes, never used others.

The questionnaire was developed in English and translated to Amharic (local) language and back to English to check for its consistency. The final questionnaire was Amharic version which was used as a self administered one. By conducting repeated revisions, the questions were made as simple as possible to be answered by the students.

In order to ensure the quality of data, the questionnaire underwent pre-testing in the same setup having similar age group as the target group, but in non-selected senior secondary schools. Vague questions that were difficult to be answered by most of the students were emphasized and corrected accordingly during the pretest. Nine supervisors who had diploma and above level of training were recruited and trained on ethical issue, confidentiality, self administered data collection technique and on how to facilitate the study.

In order to maintain confidentiality of the questionnaire sitting arrangement of the male students was considered. All eligible boys were called and made to sit in prior arranged rooms. Each student took a single seat with sparse arrangement of chairs and desks. No names or identifiers were included on the questionnaire. Each student was given a questionnaire and an envelope. Students were instructed to seal the filled questionnaire and leave it in collecting box that was prepared. The above procedures were intended to ensure confidentiality and avoid possibilities of immediate handling of filled questionnaires by supervisors.

### Data analysis

After sorting data and performing quality control for completeness and consistency, data werre coded and entered in to a computer and validated for consistency using EPI version 6 statistical packages and analysis was done using SPSS version 15.0. The data were cleaned using tabulation, frequency and sorting for outliers and accidentally entered data. Analysis of data were made by tabulation of the dependent variable against the independent variables. The dependent variables for the study were life time completed rape and sexual coercion.

Statistical test using odds ratio and 95% confidence interval was applied. Independent variables that had borderline association with the dependent variable were entered into a model to look for confounding effect in a binary logistic regression. Finally results were presented using frequency tables, ratios and figures as appropriate.

Ethical approval of the study was obtained from Institutional Review Board (IRB) of Medical Faculty Addis Ababa University. Schools’ officials at different levels were contacted, and permission was secured. Informed written consent was obtained from participants in the study. In order to assure confidentiality of the responses, names or identifiers were not written on the questionnaire. In addition students were asked not to sign on the consent form but mark (X) to confirm their decision to participate. A counselling service was provided for those who demand to obtain the services by a counselling institution, which was a local nongovernmental organization that worked specifically on sexual abuse in Addis Ababa. An experienced counsellor was recruited from the counselling institution to assist during the data collection period.

## Results

Out of the expected 884 participants, a total of 872 male students participated in the study while 12 declined to participate. Forty two respondents were excluded because of grossly incomplete and inconsistent response and this makes a response rate of 93.9%. Therefore the analysis was made on 830 completed questionnaires.

About 84% of the students were from government schools. The mean age of the study subjects was 18.66 ± 1.06 SD years. Educationally, about 56% were from grade 11 and 69% were Orthodox by religion. About 21% and 36.4% were from Oromo and Amhara ethnic group, respectively and 77% of the students were living with both parents while the rest live with their friends, relatives, alone or with others (Table 1).

**Table 1:** **Socio**-**demographic characteristics of male high schools students Addis Ababa**, **June**, **2009** (**n**=**830**)

**Character**	**No**	**%**
School type		
Government	695	83.7
Private	43	5.2
Religious/Mission	92	11.1
Age		
18	502	60.5
19	189	22.8
≥20	139	16.7
Mean ±SD	18.66±1.06	
Educational status		
11th	461	55.6
12th	369	44.5
Religion		
Orthodox	573	69.0
Muslim	175	21.1
Protestant	69	8.3
Others	10	1.2
Ethnic		
Oromo	177	21.3
Amhara	302	36.4
Tigray	79	9.5
Gurage	199	24.0
Others	78	8.7
Lives with		
Both parents	635	76.5
Friends	12	1.4
Relative	146	17.6
Alone	28	3.4
Others	9	1.1

About 47.3% and 59.4% of the students reported that their fathers and mothers were illiterate, respectively. Similarly about two in three came from a family having 5 to 9 members. 62% of the students had parents who were married while the others had divorced /separated parents or both were not alive at the time of the survey (not shown in table).

Reported sexual history and reason for sexual initiation, of the total respondents, 248(29.9%) reported that they were sexually active. The mean age at first sexual intercourse was 16.58 years with a standard deviation of 2.44 years. Two hundred thirty two (93.5%) reported to have had vaginal sexual intercourses, 12 (5%) and 9 (3.6%) reported to have had anal and oral sexual intercourses, respectively. Of the total respondents, 580 (69.9%) reported they watch pornography (not shown in the table).

In this study, life time prevalence of completed rape among male students was 4.3% with 95% confidence interval between 2.95% and 5.72%. This prevalence in the last 12 months was 5/1000 with 95% CI 0.8 to11.3 per 1000. Similarly, life time prevalence of sexual harassment experienced by male students was 68.2% with a 95% CI ranging between 65.02% and 71.36% (Table 2).

**Table 2:** **Life time and 12 months prevalence of sexual coercion among male high school students Addis Ababa**, **June**, **2009**

**Sexual coercion**	**N**^**o**^	**%**	**95% CI**
Completed rape			
Life time	36	4.3	(2.95, 5.72)
12 month	5	0.5	(0.08, 1.13)
Attempted rape			
Life time	72	8.7	(6.76, 10.59)
12 month	14	1.7	(0.81, 2.56)
Harassment			
Life time	566	68.2	(65.02, 71.36)
12 month	178	21.4	(18.65, 24.24)
Sexual coercion			
Life time	77	9.3	(7.30,11.25)
12 month	14	1.7	(0.81, 2.56 )

**Figure 1:**
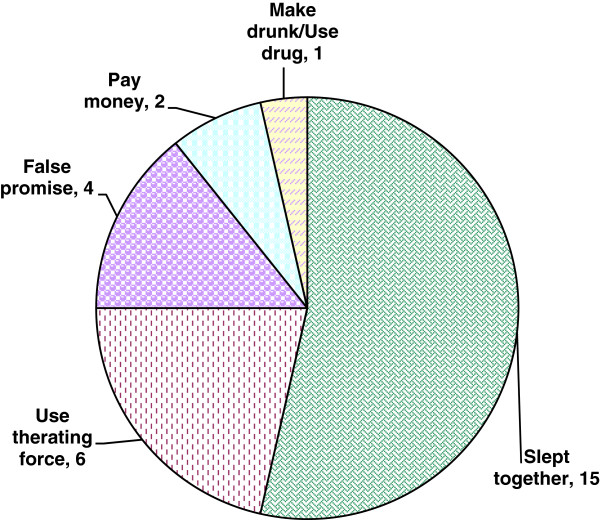
**Reported conditions during rape of male high school students**, **Addis Ababa**, **June 2009 (n= 28).**

When victims of completed rape were asked conditions during the act of rape, majority (15 of the 28), responded that they were sleeping together, and for the 6 of the 28 victims, perpetrators used threatening force (Figure 1).

When victims of rape were requested about the perpetrator, 20 (66.7%) of the perpetrators were known by the victims. Out of the students who experienced rape, for 5 (23.8%) of the victims happened in their own house and for 8 (38.1%), it was in rapist house. Twelve (52.5%) of the victims didn^’^t share the adverse act to anyone. Victims of rape were asked why they did not report to any body and keep it secret. Ten (45.5%) did not know what to do, 6 (27.3%) of the victims, they were frightened of the perpetrator and the remaining reported fear of stigma (Table 3).

**Table 3:** **Perpetrator and condition during rape of male high school students of Addis Ababa**, **June**, **2009** (**n**=**30**)

**Characteristics**	**N**^**o**^	**%**
Perpetrators		
Known	20	66.7
Unknown	10	33.3
Place of perpetrator		
In victims house	5	23.8
In rapist house	8	38.1
Hotel	4	19.0
School	4	19.0
Sharing the event to		
No body	12	52.2
Friend	7	30.4
Relatives	3	13.0
Health worker	1	4.0
Reason for not reporting		
Do not know what to do	10	45.5
Afraid of perpetrators	6	27.3
Fearing stigma	5	22.7
Afraid of parents	1	4.5

* Sample size varies due to missing responses. Analysis was done for valid cases.

**Figure 2:**
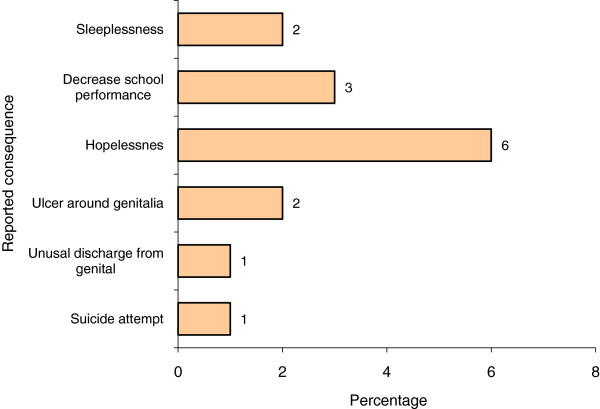
**Reported consequence on victims of sexual abuse of male high school students**, **Addis Ababa**, **June**, **2009 (n=16).**

Among the 36 boys who reported to experience completed rape, hopelessness (6) and decreased school performance (3) were reported as consequences of the sexual abuse (Figure 2).

Binary logistic regression was done to assess factors associated with completed rape and sexual coercion particularly with sociodemographic variables of the respondents and their family.

The chance of experiencing completed rape during life time was more than two-fold higher among 11th grade attendees than the 12th grade (OR = 2.15; 95% CI; 1.02, 4.51). However, there was not statistical difference in experiencing sexual coercion between 11th & 12th grade students. The odds of experiencing completed rape in life time was higher among students who live with others (OR = 2.26; 95% CI; 1.09, 4.70), than students living with their parents. Similarly the chance of experiencing sexual coercion in their life time was statistically significantly higher among students who live alone (OR = 3.34; 95% CI; 1.29, 8.62) and students who live with others (OR = 1.95; 95% CI; 1.15, 3.32) than students living with their parents. However, there was not statistically significant difference in experiencing completed rape or sexual coercion in life time by difference in school type, age, father or mother^’^s education, family size and monthly house hold income of the students (Table 4).

**Table 4:** **Comparison of life time completed rape and sexual coercion by socio**- **demographic characteristics of the study participants and their parents**, **Addis Ababa**, **June**, **2009**

**Characteristics**	**Completed rape lifetime**			**Sexual coercion**		
	**No**	**(%)**	**OR(95%CI)**	**No**	**(%)**	**OR(95%C.I)**
School type						
Government	695	32 (4.6)	1	695	68 (9.8)	1
Private	43	1 (2.3)	0.49 (0.06, 3.70)	43	1 (2.3)	0.22 (0.03, 1.62)
Religious	92	3 (3.3)	0.70 (0.21, 2.33)	92	8 (8.7)	0.88 (0.41, 1.89)
Age						
≥20	502	22 (4.4)	1	502	41 (8.2)	1
19	189	7 (3.7)	0.84 (0.35, 2.07)	189	19 (10.1)	1.26 (0.71, 2.23)
18	139	7 (5.0)	1.16 (0.48, 2.77)	139	17 (12.2)	1.57 (0.86, 2.85)
Educational status						
11th	461	26 (5.6)	2.15 (1.02, 4.51)	461	49 (10.6)	1.45 (0.89, 2.36)
12th	369	10 (2.7)	1	369	28 (7.6)	1
Lives with						
Both parents	635	21(3.3)	1	635	48 (7.6)	1
Alone	28	3(10.7)	3.50 (0.98, 12.5)	28	6 (21.4)	3.34 (1.29, 8.62)
Others	167	12(7.2)	2.26 (1.09, 4.70)	167	23 (13.8)	1.95 (1.15, 3.32)
Father education						
< Secondary	463	21( 4.5)	1	463	47 (10.2)	1
≥ Secondary	342	15(4.4)	0.96 (0.49, 1.90)	342	30(8.8)	0.85 (0.53,1.38)
Mother education						
< Secondary	463	27 (4.7)	1	579	58(10.0)	1
≥ Secondary	342	9 (3.9)	0.83 (0.38, 1.78)	232	19(8.2)	0.80 (0.47,1.38)
Family size						
<5	180	8 (4.4)	1	180	15 (8.3)	1
5-9	543	24 (4.4)	0.99 (0.44, 2.25)	543	48 (8.8)	1.06 (0.58, 1.96)
≥ 10	78	3 (3.8)	0.86 (0.22, 3.33)	78	8 (10.3)	1.26 (0.51, 3.10)
Monthly HH income						
<350	155	8 (5.2)	1	155	16 (10.3)	1
350-600	133	3 (2.3)	0.42 (0.11, 1.63)	133	8 (6.0)	0.56 (0.23, 1.34)
> 1200 Birr	248	15 (6.0)	1.18 (0.49, 2.86)	248	26 (10.5)	1.02 (0.53, 1.96)
Don’t know	294	10 (3.4)	0.65 (0.25, 1.67)	294	27 (9.2)	0.88 (0.46, 1.69)

To examine independent main effect of variables associated with the dependent variable, potential confounders were included in the model. The likelihood of experiencing completed rape was still higher to a statically significant level among students who live with others (AOR = 2.20; 95% CI; 1.04, 4.68).

Similarly, the chance of experiencing sexual coercion was still significantly higher among students who live alone (AOR = 2.87; 95% CI; 1.07, 7.66) and among students who live with others (AOR =1.80; 95% CI = 1.04, 3.11). However, the chance of experiencing sexual coercion was not associated with other variables included in the model. In the same way, the chance of experiencing completed rape was significantly different neither between 11th & 12th grade nor among students who live alone and living with their parents (Table 5).

**Table 5:** **Comparison of selected factors by completed rape and sexual coercion of male high school students**, **Addis Ababa**, **June**, **2009**

**Characteristics**	**Completed rape lifetime**		**Sexual coercion**	
	**Crude**	**Adjusted**	**Crude**	**Adjusted**
	**OR (95%CI)**	**OR (95%CI)**	**OR (95%CI)**	**OR (95%CI)**
Educational status				
11th	2.15 (1.02, 4.51)	2.02 (0.95, 4.30)	1.45 (0.89, 2.36)	1.42 (0.8, 2.34)
12th	1	1	1	1
Lives with				
Both parents	1	1	1	1
Alone	3.50 (0.98, 12.5)	3.64 (0.97, 13.7)	3.34 (1.29, 8.62)	2.87 (1.07, 7.66)
With others	2.26 (1.09, 4.70)			
	2.20 (1.04, 4.68)	1.95 (1.15, 3.32)	1.80 (1.04, 3.11)	
Age (years)				
≥20	1	1	1	1
19	0.84 (0.35, 2.07)	0.80 (0.33, 1.95)	1.26 (0.71, 2.23)	1.20 (0.67, 2.16)
18	1.16 (0.48, 2.77)	0.94 (0.37, 2.35)	1.57 (0.86, 2.85)	1.27 (0.68, 2.40)
Receiving money				
No	1	1	1	1
Yes	1.33 (0.67, 2.64)	1.14 (0.57, 2.30)	1.52 (0.94, 2.47)	1.31 (0.80, 2.16)
Slept away of home				
Never	1	1	1	1
Sometimes	1.37 (0.66, 2.84)	1.34 (0.64, 2.82)	1.35 (0.80, 2.26)	1.33 (0.79, 2.26)

*AOR=adjusted for educational status, Living condition, Age group, Receiving money, slept away of home.

## Discussion

From the total 872 male students sampled from nine governmental and nongovernmental high schools, 95.2% participated in the study. This is relatively a high response rate indicating that even if it was taboo in Ethiopian culture, male students like female students are willing to answer questions related to sexual violence. This could be due to the result of organized data collection, supervision and assurance of confidentiality.

Studies of sexual abuse among adolescents varied widely in definitions and methods used. In this study the life time prevalence rate of completed rape of male high school students of Addis Ababa was 4.3%. A study conducted in *Marakato* area of Addis Ababa has indicted that sexual abuse of male street children was as high as 28.6% [10]. However, the study mentioned above selected groups that were street male children and the coverage was limited to a large business and market area in Addis Ababa. On the other hand our findings were comparable to other reports elsewhere. For instance, the prevalence of sexual abuse of high school students of California was reported to be 4.8% [11]. It was also consistent with the results from a chart review presented at a medical clinic of Cameroon boys where the prevalence was 4.8% [12]. In the contrary, our finding was slightly lower than the prevalence among high school male students in Cape Town, which was 5.8% [13]. It was also much lower than the study done in the University of Massachusetts at Boston among male students using a self administered report of 11% [14]. It was also lower than a finding from Nigeria indicating, 15% of young females and 8% of young males reported a forced penetrative sexual experience [15].

This lower prevalence of completed rape in our study compared to other countries could be due to cultural difference in revealing the event. Or, it might also be due to fear of negative social responses towards men having sex with men. As reported by the study participants, among the reasons for not reporting incidents, the majority did not know what to do, feared perpetrators and possibilities of stigma. Hence, there might be a tendency of keeping it secret than telling it to somebody else. Ethiopia is one of the developing countries with traditional societal values expressed in terms of conservative views and practices related to children, families, gender and sexual relationships. The deep-rooted cultural and religious experiences and ties within families and communities might have a role either in protecting societal values or leading to withholding information that seem to be an unacceptable by peers, parents and other people around.

In this study those male students living alone and those living with other relatives or friends were at higher risk of experiencing sexual coercion. Similarly, male students who live with other relatives or friends had experienced completed rape in their life time than students who live with their parents. This finding is comparable to a study conducted in Soweto, that family structure is significantly related to rape where the finding was those who lived with a single parent and lived with others were more likely to be victims of sexual abuse than those living with both biological parents [13]. Possible explanation for such increase of experiencing sexual abuse of boys among those not living with their biological parents may be due to their exposure to sexual coercion and other forms of sexual abuse from opportunistic perpetrators. This may indirectly indicate the relevance of parental monitoring in terms of limiting exposure to rape and sexual coercion. Since Addis Ababa is the capital city of Ethiopia and a diplomatic center, modernization and changes in life style could affect family structure and interaction among family members including disharmony, marriage dissolution and others that affect child rearing. Moreover, the impact of HIV and AIDS and the level of orphanage in the last two decades might have some contribution in compelling children to be raised and helped by relatives and other members of the society. In our study we did not include a question that indicates who the perpetrator was. This may mask our understanding of where the perpetrators are located including close relatives and others though living with parents was found to be protective.

In our study, there was no statistically significant association between completed rape and watching pornography. This may be explained by the small number of victims in our study.

We did not find statistically significance association between sexual coercion and types of schools by ownership (government, private). This may be due to the small number of positive responses for sexual coercion in the sample. However, a study conducted in Addis Ababa has shown that attending missionary schools increased the likelihood of experiencing rape by more than two folds. In the same study, students of public schools were found more likely to experience rape than students of private schools [16].

Among the consequence of completed rape, 37% had experienced hopelessness and 18.8% had experienced problem of sleep disturbance. This was consistent with studies indicating that sexual abuse of boys and girls to have more significant emotional and behavioral problems than non-abused counterparts [17]. This may be an area of intervention where students, parents and community need to be aware of early identification of problems and make appropriate consultations.

One of the limitations of this study was that we did not know the percentage of boys 18 years and older who were attending senior secondary schools in Addis Ababa. This might affect its representativeness for the age group in the city. On the other hand, Addis Ababa is the capital city of Ethiopia and most children at the specified age group are expected to attend senior secondary schools. Moreover, the generalizability of the study was limited to those who were attending regular senior secondary schools at the time of the survey.

## Conclusions

In conclusion, this study has revealed that sexual abuse of boy students in Addis Ababa is common with a life time prevalence of rape and sexual harassment of 4.3%, and 68.2%, respectively. Living alone and not living with both parents are important factors that are related to sexual abuse of males.

We recommend sexual abuse of boy students needs due attention by schools, parents and other concerned bodies. Designing a program to fight against forcible sexual abuse should focus not only on girls but also on boys not living with their parents. It is also recommended to conduct further studies to help decision makers recognize the concerns of sexual abuse on male children.

## Competing interests

We the authors declare that we have no competing interests.

## Authors’ contributions

RH: designed, conducted and analyzed the data as part of her thesis work. NK: assisted and supervised in the design of the study and conducted analysis of the data. GK: assisted in the design of the study, conducted critical review and write up of the manuscript. All authors read and approved the manuscript.

## Pre-publication history

The pre-publication history for this paper can be accessed here:

http://www.biomedcentral.com/1472-698X/13/24/prepub
